# The crosstalk between STAT3 and p53/RAS signaling controls cancer cell metastasis and cisplatin resistance via the Slug/MAPK/PI3K/AKT-mediated regulation of EMT and autophagy

**DOI:** 10.1038/s41389-019-0165-8

**Published:** 2019-10-09

**Authors:** Fan Liang, Chunxia Ren, Jingshu Wang, Shuoer Wang, Lina Yang, Xianghui Han, Yaping Chen, Guoqing Tong, Gong Yang

**Affiliations:** 1 Department of Obstetrics and Gynecology, The Fifth People’s Hospital of Shanghai, Fudan University, Shanghai, 200240 China; 2 Central Laboratory, the Fifth People’s Hospital of Shanghai, Fudan University, Shanghai, 200240 China; 3 Center for Reproductive Medicine, Shuguang Hospital Affiliated to Shanghai University of Traditional Chinese Medicine, Shanghai, 200120 China; 4 Institute of Chinese Traditional Surgery, Longhua Hospital Affiliated to Shanghai University of Traditional Chinese Medicine, Shanghai, 200032 China; 5 Cancer Institute, Fudan University Shanghai Cancer Center, Shanghai, 200032 China; 6 Department of Oncology, Fudan University Shanghai Medical College, Shanghai, 200032 China

**Keywords:** Ovarian cancer, Cancer therapeutic resistance

## Abstract

Chemoresistance has been the biggest obstacle in ovarian cancer treatment, and STAT3 may play an important role in chemoresistance of multiple cancers, but the underlying mechanism of STAT3 in ovarian cancer chemoresistance has long been truly illusive, particularly in association with p53 and RAS signaling. In this study, by using wild type, constitutive active, and dominant negative STAT3 constructs, wild-type p53, and RAS-mutant V12, we performed a series of in vitro and in vivo experiments by gene overexpression, drug treatment, and animal assays. We found that phosphorylation of STAT3 Y705 but not S727 promoted cancer cell EMT and metastasis through the Slug-mediated regulation of E-cadherin and Vimentin. The phosphorylation of STAT3 at Y705 also activated the MAPK and PI3K/AKT signaling to inhibit the ERS-mediated autophagy through down-regulation of pPERK, pelf2α, ATF6α, and IRE1α, which led to increased cisplatin resistance. Induction of wild type p53 in STAT3-DN-transfected cells further diminished the chemoresistance and tumor growth through the upregulation of the MAPK- and PI3K/AKT-mediated ERS and autophagy. Introduction of STAT3-DN deprived the RAS^V12^-induced ERS, autophagy, oncogenicity, and cisplatin resistance, whereas introduction of p53 in STAT3-DN/RAS^V12^ expressing cells induced additional tumor retardation and cisplatin sensitivity. Thus, our data provide strong evidence that the crosstalk between STAT3 and p53/RAS signaling controls ovarian cancer cell metastasis and cisplatin resistance via the Slug/MAPK/PI3K/AKT-mediated regulation of EMT and autophagy.

## Introduction

Ovarian carcinoma is the most lethal gynecological malignancy, which ranks the fifth leading cause of cancer death among women^1,2^. Although the standard therapy of cytoreductive surgery followed by chemotherapy with paclitaxel and platinum has improved the clinical outcome, the 5-year survival rate is still less than 50% most likely due to high chemoresistance of recurrent cancer cells^2,3^. Therefore, understanding and targeting the chemoresistant mechanism of cancer cells would potentially improve the treatment efficacy and survival of ovarian cancer patients.

Autophagy is a cellular evolutionarily conserved process in eukaryotes, from yeast to mammals^4^. When cells encounter stresses from extracellular stimuli, autophagy could be induced to form autophagosomes to initiate a self-eating cellular process through which harmful components could be degraded and cellular homeostasis could be maintained^4,5^. Autophagy may contribute to either chemoresistance or chemosensitivity through different signaling contexts under specific conditions in different cancer types^6,7^. Recent literatures have also endorsed the growing evidence that autophagy could be differentially regulated by signal transducer and activator of transcription 3 (STAT3): nuclear STAT3 may finely regulate autophagy via the transcriptional regulation of several autophagy-related genes; whereas cytoplasmic STAT3 can inhibit autophagy by sequestering EIF2AK2 and interacting with other molecules such as FOXO1 and FOXO3, and the mitochondrial STAT3 suppresses the oxidative stress-induced autophagy and reserves mitochondria from being degraded by mitophagy^8^. Therefore, the signaling pathways involved in the STAT3-associated autophagy are quite intricate particularly in terms of cancer cell chemoresistance.

STAT3 is a cytoplasmic transcription factor that mediates cytokine and growth factor signaling^9^. Following phosphorylation and activation, STAT3 dimers translocate into the nucleus, where they bind to specific DNA response elements in the promoter of target genes and activate their expression^10^. In normal physiological conditions, STAT3 is activated temporarily from a few minutes to several hours; however, in human cancer cells, STAT3 can be constitutively activated^9,11^. Early studies reported that inhibition of the constitutively activated Stat3 by using the Janus Kinase-selective inhibitor (AG490) suppresses ovarian cancer growth^12^, and disruption of constitutively activated STAT3 overcomes ovarian cancer cisplatin resistance^13^. STAT3 was constitutively activated at Tyr 705 in cancer cells derived from ovarian cancer patient ascites, resulting in platinum-based chemoresistance^14^. However, the underlying mechanism of STAT3 in association with autophagy and chemoresistance in ovarian cancer is quite ambiguous.

In this study, we demonstrate that the constitutively activated STAT3 promotes EMT and inhibits the ERS-associated autophagy, leading to ovarian cancer cell proliferation, metastasis, and cisplatin resistance through a crosstalk with p53 and RAS signaling molecules including Slug, MAPK and PI3K/AKT/mTOR.

## Materials and methods

### Cell lines and cell culture

Human ovarian epithelial cancer cell lines SKOV3, OVCA429, HEY, A2780, and lentivirus packaging cells (HEK293T) were purchased from American Type Culture Collection (ATCC, USA) or maintained in our laboratory and were recently identified by STR profiling by Genetic Testing Biotechnology (Suzhou, China). SKOV3, OVCA429, HEY, and A2780 cells were maintained in Roswell Park Memorial Institute (RPMI) 1640 medium, HEK293T cells were cultured with Deulbecco’s Modified Eagle Medium (DMEM). Both media were supplemented with 10% fetal bovine serum, 2 mM l-glutamine, penicillin (100 units/ml), and streptomycin (100 μg/ml). Cells were incubated at 37 °C in an atmosphere of 5% CO_2_ and 95% air.

### Cell transfection and viral infection

SKOV3-p53 tet on cells (SKOV3^T^) expressing the p53-tet on system were constructed following the Lenti-X^TM^ Tet-On Advanced Inducible Expression System user manual (Clontech 632162). The resulting cells were selected with neomycin (500 μg/ml) and hygromycin (2 μg/ml) for 7-14 days. To establish SKOV3^T^cell lines expressing HRAS-V12, the full length RAS^V12^ cDNA was amplified from previously established plasmids in our lab using the primers 5′-CGCggatccATGACCGAATACAAGCTTGTTG -3′ (forward; lower case letters represent the *Bam*HI site) and 5′-TGAT*ctcgag*TCAatggtgatggtgatgatgGGAGAGCACACACTTGCAGCTCA-3′ (reverse; italic lower case letters represent the*Xho*I site; bold lower case letters indicate the His tag), digested with *Bam*HI and *Xho*I, and inserted into the retrovirus vector pBabe-zeo. The correct plasmids were confirmed by sequencing^15^. Constitutive activated STAT3 (STAT3-C), wild-type STAT3 (STAT3-WT), and dominant negative STAT3 (STAT3-DN) containing HA-tag plasmids were constructed into lentiviral vector pCDH-CMV-MCS-EF1- puro with *Eco*RI and *Bam* HI from the original plasmids purchased from Addgene. Viruses produced from HEK293T cells were collected to infect target cells and to establish OVCA429-STAT3-C, OVCA429-STAT3-WT, SKOV3-STAT3-DN, SKOV3-p53, SKOV3-p53-V12, and SKOV3-p53-V12-DN cell lines, using the previously published methods^16^. Corresponding control cell lines were made by infection of viruses expressing empty vectors. The positive clones were selected with puromycin (1.5–2.0 μg/mL) or zeocin (5–10 µg/ml) for 10–14 days. The resulting cells were used for following experiments without addition of puromycin or zeocin.

### Cell proliferation

Cells were detached using trypsin and washed twice with PBS. 2 × 10^3^ cells per well were seeded in 96-well culture plates (Corning Inc., Corning, NY) in 100 μl medium and cultured for 1, 2, 3, 4, and 5 days. Cell growth was detected using 5 mg/mL MTT solution (sigma) according to the manufacturer’s instructions. The OD at 490 nm was quantified using a Tecan Infinity 200PRO multi-well plate reader (Tecan Ltd., Switzerland). The assay was independently repeated three times.

### Plate colony formation assay

According to the previously published method^17^, cells stably expressing STAT3-C, STAT3-WT, STAT3-DN and their corresponding controls were used to perform plate colony formation assay. Briefly, cells were suspended in 1640 containing 10% FBS and seeded in six-well culture plates (200 cells per well). Triplicate cultures of each cell line were maintained for 14–28 days at 37 °C in a 5% CO_2_ atmosphere, and fresh medium was fed every 7 days. After 20 days, colonies could be observed directly with the unaided eye. The colonies were fixed with 4% paraformaldehyde for 15 min and stained with crystal violet for 15 min at ambient temperature. After washing twice with PBS, the colonies were viewed and counted under a microscope at ×40 magnification. Only clearly visible colonies (diameter > 50 µm) were counted.

### Cell invasion and migration assay

To identify cell invasion ability, we used a high throughput screening multi-well insert 24-well two-chamber plate (BD Biosciences, San Jose, CA), with an 8-μm (pore size) polycarbonate filter between chambers. 2.5 × 10^4^ cells of cells expressing STAT3-C, STAT3-WT, STAT3-DN and their corresponding controls were placed into the upper chamber and permitted to invade at 37 °C for 48 h toward a lower reservoir containing medium and coated with Matrigel (BD Biosciences). The chambers were then fixed in 100% methanol for 30 min and stained with crystal violet for 10 min. The invasive cells, which passed through the membrane, were counted at ×200 magnification with five representative fields under a microscope. All the above assays were repeated in triplicate.

Scratch assay was performed to examine cell migration speed. Cells were incubated in six-well plate overnight to yield monolayer confluence. By scratching with a pipette tip and photographing immediately (time 0), 24 h’ later and 48 h’ later, the distance migrated by the cell monolayer to close the scratch area during the time period was observed and measured. The ratio of the cell migration distance at 48 h to that at 0 h was analyzed as the migration index. The assay was carried out in triplicate and repeated three times.

### Cell treatment and cell viability assay

Cisplatin was purchased from Haosen pharmaceutical company (Jiangsu, China). Stock concentration of cisplatin was 5 mg/ml and the concentration used to treat ovarian cancer cell lines was 0–100 μM. Cells were detached using trypsin and washed twice with PBS. 4 × 10^3^ cells of SKOV3-STAT3-DN per well and corresponding control cells were seeded in 96-well culture plates (Corning Inc., Corning, NY) in 100 μl medium. 3 × 10^3^ cells of OVCA429-STAT3-C and OVCA429-STAT3-WT and OVCA429-PCDH-Vector cell lines per well were seeded in 96-well culture plates. 4 × 10^3^ cells of HEY, SKOV3, A2780 and OVCA429 cell lines per well were seeded in 96-well culture plates. Then medium containing different concentrations of cisplatin was added and cultured for 48 h. Cell viability was detected, using 5 mg/mL MTT solution (Sigma-Aldrich product) according to the manufacturer’s instructions. The OD at 490 nm was quantified using a Tecan Infinity 200PRO multi-well plate reader (Tecan Ltd., Switzerland). Then IC50 value was calculated. Cell treatment was performed by IL-6 (20 umol/L) and HO-3867 (10 umol/L) for 2 h according to the method described elsewhere^18^.

### Immunofluorescence staining

Cells were plated on glass slides in 24-well culture plates at a concentration of 2 × 10^4^ cells/well for 24 h. Thereafter, the cells were fixed with a 4% formaldehyde solution in PBS, permeabilized with 0.5% TritonX-100 in PBS, stained with the primary antibody LC3B overnight, and labeled with anti-rabbit IgG conjugated with Alexa Fluor 594. The cells were counterstained with DAPI and observed under a fluorescence microscope.

### Preparation of cell extracts and immunoblotting

We prepared cell lysates at 75% of confluence using 500 μL of radioimmunoprecipitation assay buffer (RIPA, 25 mmol/L Tris–HCl at pH 7.6, 150 mmol/L NaCl, 1% Nonidet P-40, 1% sodium deoxycholate, and 0.1% sodium dodecyl sulfate). For cisplatin-treated SKOV3-STAT3-DN cells and corresponding control cells, OVCA429-STAT3-C, OVCA429-STAT3-WT, and control cells, we plated the same number of cells in a 60 mm culture dish. After adherence, the cells were treated with cisplatin at the corresponding IC50 concentration for 48 h. Protein samples were prepared by lysis with RIPA buffer and the protein concentrations of the lysates were determined with a Bio-Rad protein assay kit (Hercules, CA). Immunoblotting analyses were performed as described previously^16^. Antibodies against the following proteins were obtained from Santa Cruz Biotechnology: p-STAT3 (S727), E-cadherin, Slug, Snail, Vimentin, p62, ATG5, BECLIN1, ATG12, GPR78/BIP, p-PERK (Thr981), HRAS, p53, ATF6α and PI3K-p85α. Antibodies against the following proteins were from Cell Signaling Technology (Danvers, MA): STAT3, p-STAT3 (Y705), HA-tag, LC3B, elf2α, pelf2α, ATF4, IRE1α, MEK1/2, p-MEK1/2, ERK1/2, p-ERK1/2, p-P38, p-AKT (Ser473), mTOR, p-mTOR, 4EBP1, p4EBP1. The secondary antibodies including anti-mouse IgG and anti-rabbit IgG linked to HRP were from Cell Signaling Technology (Danvers, MA). The immunoblots were visualized using the enhanced chemiluminescence reagent (Thermo Scientific, USA).

### Xenograft tumors in nude mice

The animal experiments were approved by the Institutional Animal Care and Use Committee of the Fifth People’s Hospital of Shanghai, Fudan University and performed following the Institutional Guidelines and Protocols. Xenograft tumors were either subcutaneously to detect tumor growth or intraperitoneally to monitor tumor metastasis of various cell lines and their corresponding controls. 5 × 10^6^ cells of each cell line mentioned above were bilaterally injected into 6-week-old female BALB/c athymic nude mice offered by Slac Laboratory Animal (Shanghai, China). Mice were randomly divided into groups. Each cell line was injected into three mice for a total of six injections. Subcutaneous tumors were measured with a vernier caliper every 3 days. When a tumor reached 1.0 cm in diameter, the mouse was sacrificed and the tumors were weighed and measured. The longest diameter “a” and the shortest diameter “b” of tumors was measured, and we calculated the tumor volume with the formula: V (in mm3) = a × b^2^ × 0.52, where 0.52 is a constant to calculate the volume of an ellipsoid. Six mice were chosen to be injected with 1 × 10^7^ cells of each cell lines intraperitoneally, and mice were observed regularly and sacrificed before natural death occurred. Tumor nodules were removed, counted, and the mice were weighed. Mouse experiments were conducted in a single blind manner.

### Statistical analysis

All data were analyzed by the Student *t*-test. *p* < 0.05 was considered statistically significant.

## Results

### Phosphorylation of STAT3 at tyrosine 705 promotes cell proliferation, and enhances cell migration and invasion through control of EMT associated markers

**Fig. 1:**
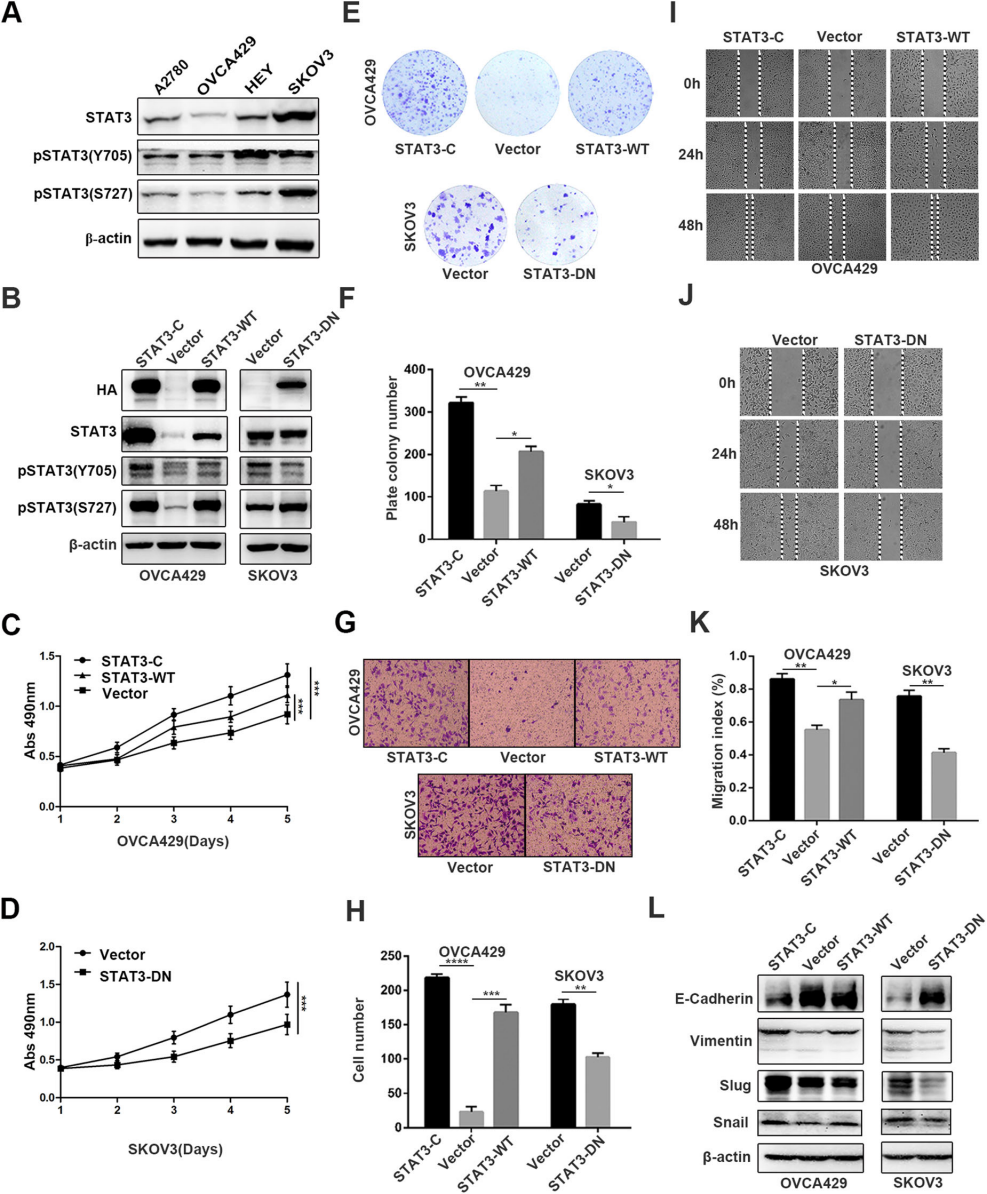
STAT3 promotes cell proliferation, invasion, and migration. **a** Detection of pSTAT3 Tyr705, pSTAT3 Ser727 expression by western blot in A2780, OVCA429, HEY, and SKOV3 cells. **b** Detection of HA-tag, STAT3, pSTAT3 Tyr705, and pSTAT3 Ser727 in constructed OVCA429 and SKOV3 cell lines. **c**, **d** Detection of cell proliferation by MTT at the indicated time points (*P* < 0.05). **e**, **f** Colony-forming capability of cancer cells at 14 days. **g** Quantitative analysis of migrated cells. **h** Detection of cell invasion by using a high throughput screening multi-well insert 24-well two-chamber plates. **i**, **j** Detection of migration speed by scratch assay. **k** Quantitative analysis of migration speed by migration index. **l** Detection of metastasis-associated proteins by western blot. β-actin was used as a loading control. *p < 0.05, **p < 0.01. Error bars = 95% CIs

To investigate the function of STAT3 in human ovarian cancer, we first detected the expression level of STAT3 and pSTAT3 Y705 and S727 in four human ovarian cancer cell lines. We found that the expression of pSTAT3 (Y705) was higher in HEY and SKOV3 cells than in A2780 and OVCA429 cells (Fig. 1a), and the expression levels of STAT3 and pSTAT3 S727 were higher in SKOV3 cells than in A2780, HEY and OVCA429 cells. Thus, SKOV3 cells were chosen to establish STAT3-DN cell line, and OVCA429 cells were chosen to generate STAT3-C and STAT3-WT cell lines (OVCA429-STAT3-C and OVCA429-STAT3-WT), corresponding control cells were infected with lentiviruses expressing PCDH empty vector. As tested by western blot, HA was detected in OVCA429-STAT3-C, OVCA429-STAT3-WT, and SKOV3-STAT3-DN cells, in which HA was tagged at the 3’ end of STAT3-C, STAT3-WT and STAT3-DN cDNAs, respectively (Fig. 1b). The expression level of STAT3 was remarkably increased in both OVCA429-STAT3-C and OVCA429-STAT3-WT cells, but was not altered in SKOV3-STAT3-DN cells, compared within their relative controls. pSTAT3 Tyr705 was markedly increased in OVCA429-STAT3-C cells and slightly increased in OVCA429-STAT3-WT cells, and was reduced in SKOV3-STAT3-DN cells. pSTAT3 Ser727 expression was increased in cells expressing STAT3-C, STAT3-WT and STAT3-DN, compared with cells expressing PCDH-Vector. We next evaluated cell proliferation rate by MTT assay. The results showed that cells expressing STAT3-C, STAT3-WT had higher ability of proliferation, while cells expressing STAT3-DN had lower ability of proliferation than did the PCDH-Vector expressing control cells (Fig. 1c, d). Consistently, a colony formation assay showed that the number of colonies formed by OVCA429-STAT3-C and OVCA429-STAT3-WT cells was significantly increased, while the colony number formed by SKOV3-STAT3-DN cells were obviously decreased compared with those formed by controls (Fig. 1e, f). These results demonstrate that the phosphorylation of STAT3 at Y705 but not S727 controls cell proliferation.

To test the effects of STAT3-C/STAT3-WT and STAT3-DN on invasion and migration, we first performed assays using a high throughput transwell with matrix gel. We found that the number of invaded cells expressing STAT3-C and STAT3-WT was highly increased, whereas the number of invaded cells expressing STAT3-DN was much reduced compared with that of control cells (Fig. 1g, h). We then detected migration speed by scratch assay and found that the migration speed of OVCA429-STAT3-C and OVCA429-STAT3-WT cells was accelerated after 24 h and 48 h culture, but the migration speed of SKOV3-STAT3-DN cells was slowed down (Fig. 1i–k). EMT (Epithelial-to-Mesenchymal Transition) is associated with cell migration and invasion, so we determined the expression of migration related proteins, including E-cadherin, Vimentin, Slug and Snail. Compared with in control cells, the expression of E-cadherin was decreased in OVCA429-STAT3-C and OVCA429-STAT3-WT cells, and increased in SKOV3-STAT3-DN cells (Fig. 1l). Vimentin, Slug, and Snail were upregulated in OVCA429-STAT3-C and OVCA429-STAT3-WT cells, and downregulated in SKOV3-STAT3-DN cells (Fig. 1l). These results suggested that the phosphorylation of STAT3 at Y705 strongly stimulates ovarian cancer cell invasion and migration possibly through the Slug/Snail-mediated upregulation or downregulation of Vimentin or E-cadherin.

### Phosphorylation of STAT3 at Y705 enhances cell resistance to cisplatin-induced apoptosis via inhibition of ER stress-mediated autophagy

**Fig. 2:**
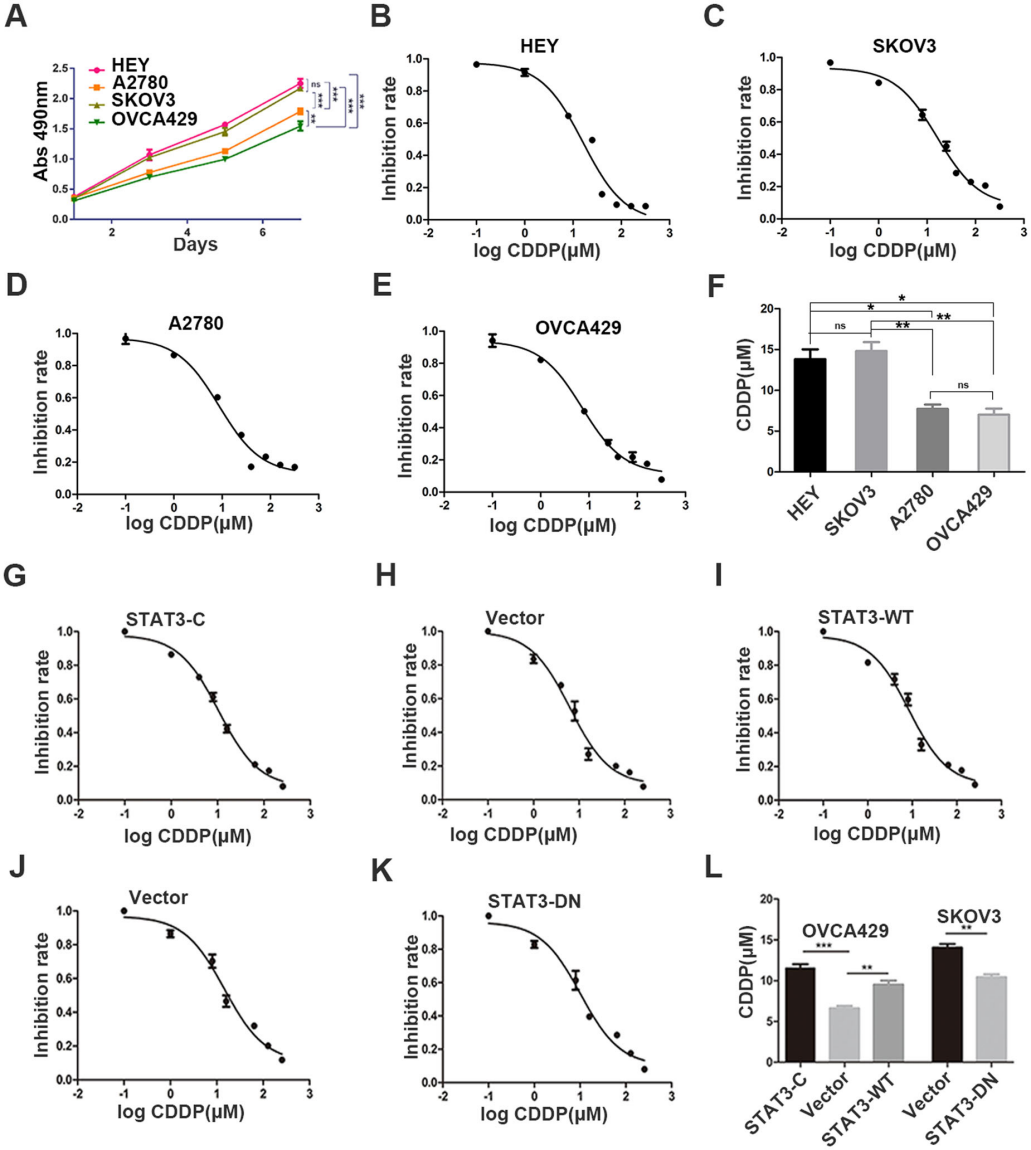
Cell proliferation and cisplatin resistance. **a** Detection of cell proliferation by MTT at the indicated time points. **b–e** Sensitivity to cisplatin tested by MTT in HEY, SKOV3, A2780, and OVCA429 cells showing the inhibition rates. **f** Quantitative analysis of IC50 values. **g**–**k** Sensitivity to cisplatin tested by MTT in OVCA429 and SKOV3 cells with or without the alteration of STAT3 activation showing the inhibition rates. **l** Quantitative analysis of IC50. *p < 0.05, **p *<* 0.01, ***p *<* 0.001. Error bars = 95% CIs

To examine the basal proliferation ability of ovarian cancer cell lines with different levels of STAT3 activation, we first detected the cell viability by MTT assay. According to Fig. 2a, HEY and SKOV3 cells yielded a higher proliferation ability than A2780 and OVCA429 cells, which was consistent with the expression levels of STAT3 at Y705 in above cell lines. Besides, we further studied the sensitivity of different cell lines to cisplatin and found that A2780 and OVCA429 cells were relatively sensitive to cisplatin with a low IC50 values when compared with HEY and SKOV3 (Fig. 2b–f). The above results indicate that the activated status of STAT3 at Y705 in ovarian cancer cell lines may play an important role in cisplatin resistance. To further investigate the effect of STAT3-C and STAT3-DN on cell sensitivity to cisplatin, we performed the same assays. The results demonstrated that cells expressing STAT3-C and STAT3-WT conferred more chemoresistance than control cells in OVCA429 series cell lines, whereas STAT3-DN promoted cell sensitivity to cisplatin treatment (Fig. 2g–l), compared with cells expressing vector.

**Fig. 3:**
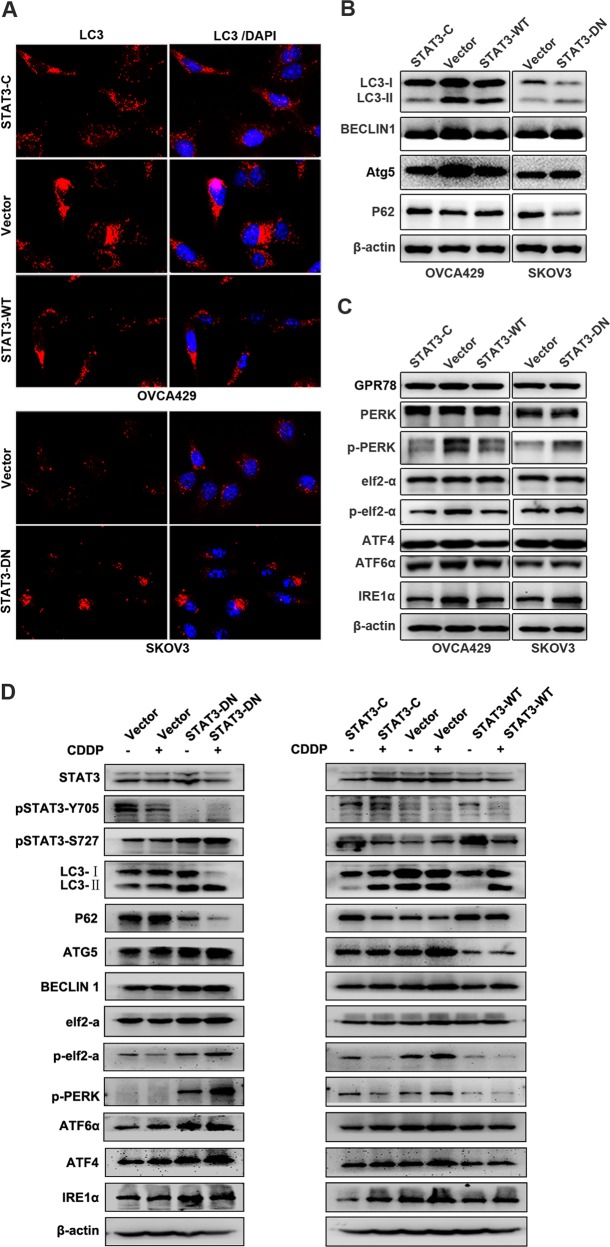
STAT3 status and cisplatin treatment alter autophagy and ER stress. **a** Detection of LC3B by immunofluorescence. **b**, **c** Analysis of autophagy-associated molecules and ER stress-associated molecules by western blot. **d** Analysis of autophagy-associated molecules and ER stress-associated molecules by western blot in different cell lines upon cisplatin treatment. β-actin was used as a loading control

Because STAT3 is associated with autophagy^8^, so we examined the effect of STAT3 status on autophagy. We found that less autophagosomes accumulated in OVCA429-STAT3-C and OVCA429-STAT3-WT cells than in OVCA429-Vector cells. Inversely, more autophagosomes were detected in SKOV3-STAT3-DN cells than in SKOV3-Vector cells (Fig. 3a). This result was further confirmed by detection of autophagy-associated proteins. As shown in Fig. 3b, the conversion of LC3-I to LC3-II, which is the autophagosome-associated form, was reduced in cells expressing STAT3-C and STAT3-WT and enhanced in cells expressing STAT3-DN, compared with in cells expressing vector. In addition, the protein levels of BECLIN1 and ATG5 were decreased in cells expressing STAT3-C and STAT3-WT and increased in cells expressing STAT3-DN. Because P62 is an autophagic inhibitor, we tested and found that P62 was either increased by STAT3-C and STAT3-WT or decreased by STAT3-DN (Fig. 3b). It has been reported that endoplasmic reticulum stress can induce or inhibit autophagy via multiple signal pathways^19^, so we detected several ER stress-associated molecules. We found that the expression of p-PERK, pelf2α, and IRE1α were downregulated in cells expressing STAT3-C and STAT3-WT and upregulated in cells expressing STAT3-DN, whereas the expression of GPR78, PERK, elf2-α, ATF4, and ATF6α was not clearly changed in terms of the STAT3 status (Fig. 3c). These data suggested that the phosphorylation of STAT3 at Y705 may confer cancer cell chemoresistance through inhibition of the ERS-mediated autophagy.

Because ovarian cancer is usually treated by cisplatin, we tested whether the above autophagy/ ERS-associated proteins are altered in cells treated with cisplatin by western blot. In comparison with control cells, the phosphorylation levels of STAT3 were different in the two cell lines. In SKOV3-Vector cells, the activation form of STAT3 at Y705 site was decreased, whereas that at S727 site was increased. In SKOV3-DN cells, the activation level of STAT3 at Y705 site was not clearly changed, but that at S727 site was increased. In OVCA429 series cells, the expression level of Y705 in STAT3-C expressing cells was not obviously changed. The activation of STAT3 at Y705 in STAT3-WT and Vector control cells was decreased, while the activation of STAT3 at S727 in all cell lines was decreased after cisplatin treatment (Fig. 3d).

Upon treatment of cells with cisplatin, the conversion rate of LC3-I to LC3-II (autophagy-related form) was remarkably enhanced in SKOV3-DN cells compared with that in control cells, and was increased in STAT3-C and STAT3-WT-expressing cells compared with in OVCA429-Vector cells, which may be due to the high levels of basal autophagy in OVCA429-Vector cells. It was consistent that the level of p62 in each group was decreased after addition of cisplatin. The upregulation of ATG5 was shown in OVCA429-Vector cells, but not in STAT3-C and STAT3-WT cells. The expression level of BECLIN1 was not clearly altered by cisplatin treatment (Fig. 3d). Thus, the activation of STAT3 at Y705 may mainly function to eliminate the cisplatin-induced autophagy in ovarian cancer cells.

We further examined some endoplasmic reticulum stress-related proteins. We found that p-PERK, pelf2α, IRE1α, ATF4, and ATF6α were markedly increased in SKOV3-DN cells after cisplatin treatment, but not in control cells. In OVCA429 series cells, p-PERK, pelf2α, IRE1α, ATF4, and ATF6α were increased in control cells compared with in STAT3-C and STAT3-WT-expressing cells. These results suggest that the cisplatin-induced endoplasmic reticulum stress may be highly inhibited by the activation of STAT3 Y705, which may reduce the sensitivity of cells to cisplatin treatment.

### MAPK and PI3K/AKT/mTOR mediate the STAT3-inhibited autophagy

**Fig. 4:**
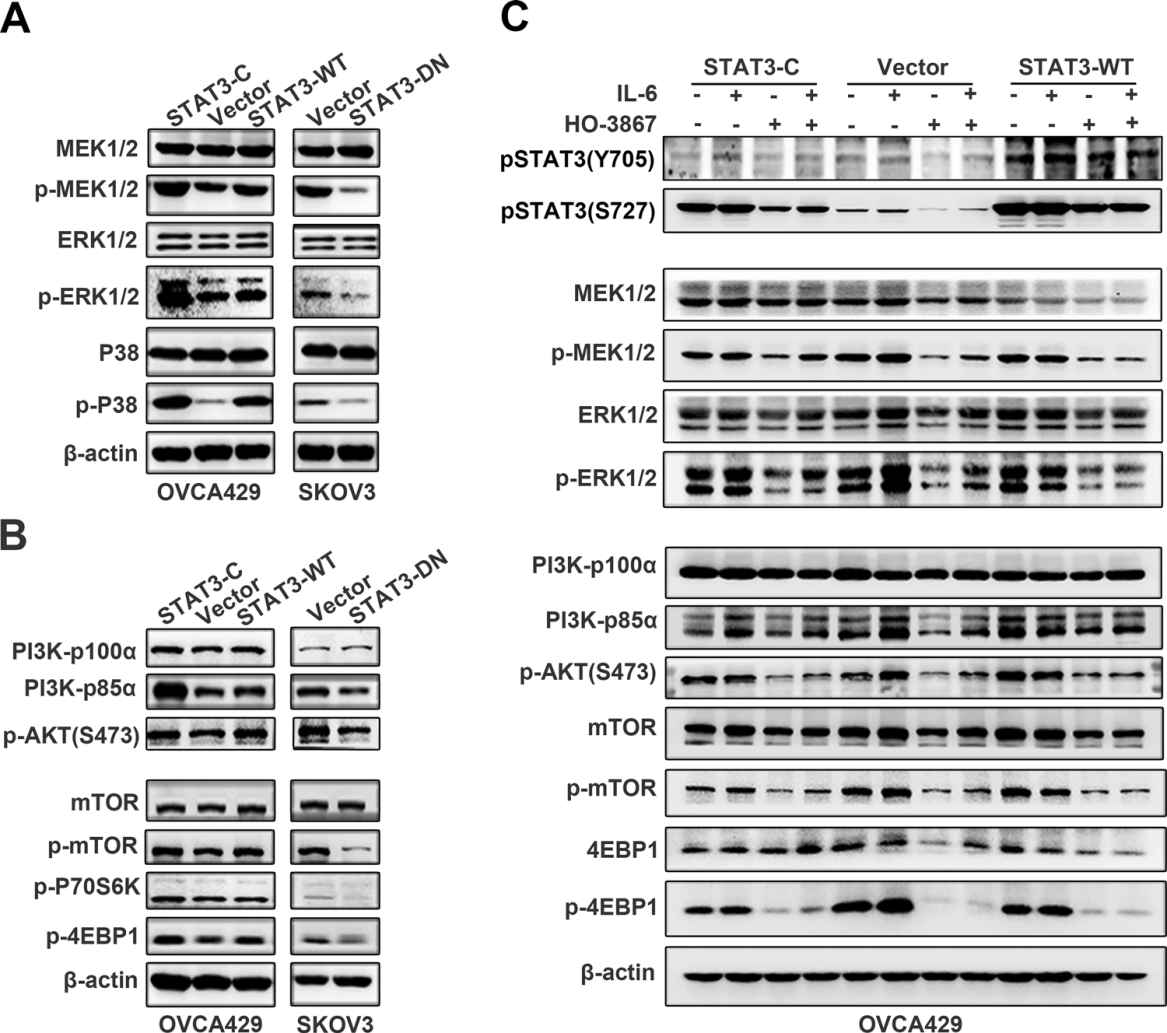
IL-6/STAT3 regulates the expression of MAPK and PI3K/AKT/mTOR. **a**, **b** Detection of MAPK (**a**) and PI3K/AKT/mTOR (**b**) signaling molecules. **c** Immunoblotting analysis of pSTAT3 (S727) and pSTAT3 (Y705) (upper panel), MEK/EKR (middle panel) and PI3K/AKT/mTOR (lower panel) with or without the treatment of IL-6 and HO-3867. β-actin was used as a loading control

To elucidate the potential mechanism of how STAT3 activation inhibited autophagy in ovarian cancer cells, we detected major members in MAPK and PI3K/AKT/mTOR signal networks by western blot. We found that MEK1/2, ERK 1/2 and P38 expression were not changed without regard to STAT3 status in all cell lines, but the phosphorylated proteins of MEK1/2 (Ser217/221), ERK1/2 (Thr202/Tyr204) and P38(Thr180/Tyr182) were either increased in STAT3-C and STAT3-WT expressing cells, or decreased in STAT3-DN expressing cells (Fig. 4a). The expression of PI3K-p85α and pAkt S473 was upregulated by STAT3-C and STAT3-WT and downregulated by STAT3-DN, whereas PI3K-p110α was intact in terms of STAT3 status (Fig. 4b upper panel). In addition, we found that the expression levels of p-mTOR and its downstream targets p-P70S6K and p-4EBP1 were consistently upregulated by STAT3-C and STAT3-WT and down-regulated by STAT3-DN cells, although the basal level of mTOR was not changed in all cell lines (Fig. 4b lower panel). These data suggested that STAT3 may inhibit ER stress-associated autophagy through the activation of MAPK and PI3K/AKT/mTOR signal pathways.

To confirm the crosstalk between STAT3 and MAPK or PI3K signaling networks, we treated STAT3-C, Vector, and STAT3-WT-expressing cells with IL-6 and STAT3 inhibitor HO3867 and examined the major molecules involved in MAPK, PI3K/AKT, and mTOR signal pathways. We found that IL-6 upregulated pSTAT3 Tyr705 but not pSTAT3 Ser727 expression in OVCA429 cells in the presence or absence of exogenous STAT3 expression, whereas treatment of cells with HO-3867 downregulated the expression of both pSTAT3 Y705 and pSTAT3 S727 (Fig. 4c upper panel). No apparent alterations of MEK1/2 and ERK1/2 were conceived in cells treated with or without IL-6 or HO3867, whereas the phosphorylated ERK1/2 (Thr202/Tyr204) but not MEK1/2 (Ser217/221) was lifted by IL-6 treatment. However, both pMEK1/2 and pERK1/2 were largely inhibited by HO3867 treatment (Fig. 4c middle panel). Moreover, PI3K-p85α and pAkt (S473) were increased by IL-6 treatment and reduced by HO3867 in all cell lines, whereas PI3K-p110α was not altered by IL-6 or HO3867 treatment The expression levels of mTOR, p-mTOR, 4EBP1, p4EBP1 were consistently promoted by IL-6 and inhibited by HO-3867 (Fig. 4c lower panel). These data together demonstrated that MAPK and PI3K/Akt/mTOR signal pathways mediate the IL-6/STAT3-inhibited ER stress and autophagy, which may lead to cancer cell cisplatin resistance.

### Activation of STAT3 promotes ovarian tumorigenesis and metastasis

**Fig. 5:**
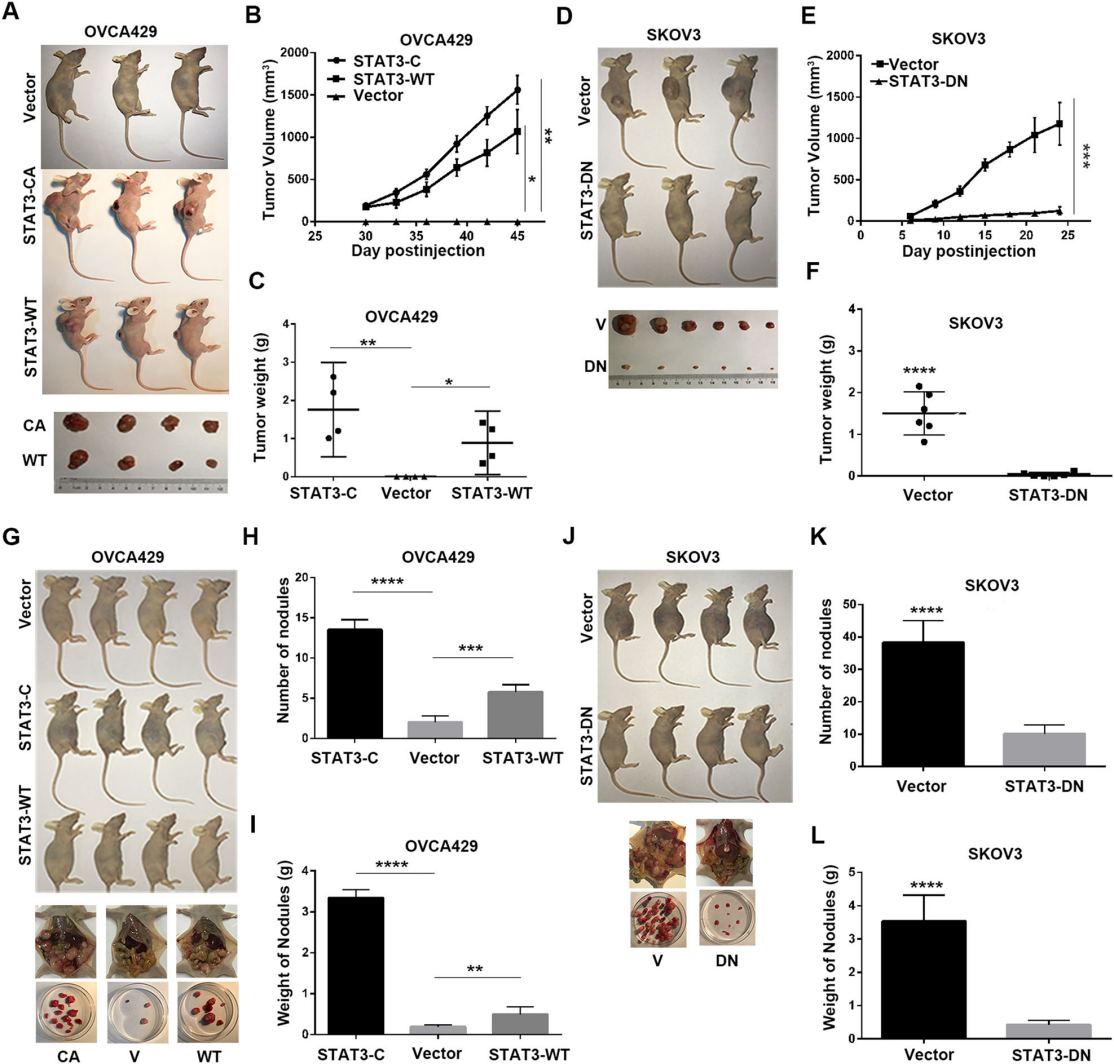
Xenograft tumor growth in mice. **a**–**c** Representative subcutaneous tumors, growth curves, and tumor weights induced by OVCA429 cells expressing vector, STAT3-CA and STAT3-WT. **d**–**f** Representative subcutaneous tumors, growth curves, and tumor weights induced by SKOV3 cells expressing vector and STAT3-DN. **g**–**i** Representative intraperitoneal tumor and nodules, nodule number, and nodule weights induced by OVCA429 cells expressing vector, STAT3-CA and STAT3-WT. **j**–**l** Representative intraperitoneal tumor and nodules, nodule number, and nodule weights induced by SKOV3 cells expressing vector and STAT3-DN. Error bars = 95% CIs. *n* = 6 mice/group. Arrows indicate metastatic nodules. ***P < 0.001, ****P < 0.0001 vs controls

To determine the in vivo effects of STAT3 on ovarian cancer tumorigenesis and metastasis, we performed animal assays with cells expressing STAT3-CA, STAT3-WT, and STAT3-DN along with those expressing empty vector. Mice were subcutaneously or intraperitoneally injected with the above cells. Tumor sizes were measured every 3 days. As shown in Fig. 5, no subcutaneous tumors were observed in mice injected with OVCA429-Vector cells after 45 days, whereas tumors from mice injected with cells expressing STAT3-CA or STAT3-WT exhibited rapid growth with large volume and heavy weight (Fig. 5a–c). Meanwhile, tumors from nude mice injected with SKOV3-STAT3-DN cells grew slower, smaller, and lighter than those derived from control cells (Fig. 5d–f). Peritoneal tumor analysis showed that the number and weight of metastatic nodules were increased by STAT3-C and STAT3-WT, but decreased by STAT3-DN transfection (Fig. 5g–l) compared with mice injected with cells expressing PCDH-Vector. Together, these results suggested that activation of STAT3 accelerates ovarian tumorigenesis and metastasis.

### p53 and RAS participate in the STAT3-induced ERS, autophagy, chemoresistance, and tumorigenesis through regulation of the PI3K/AKT and MAPK signaling

**Fig. 6:**
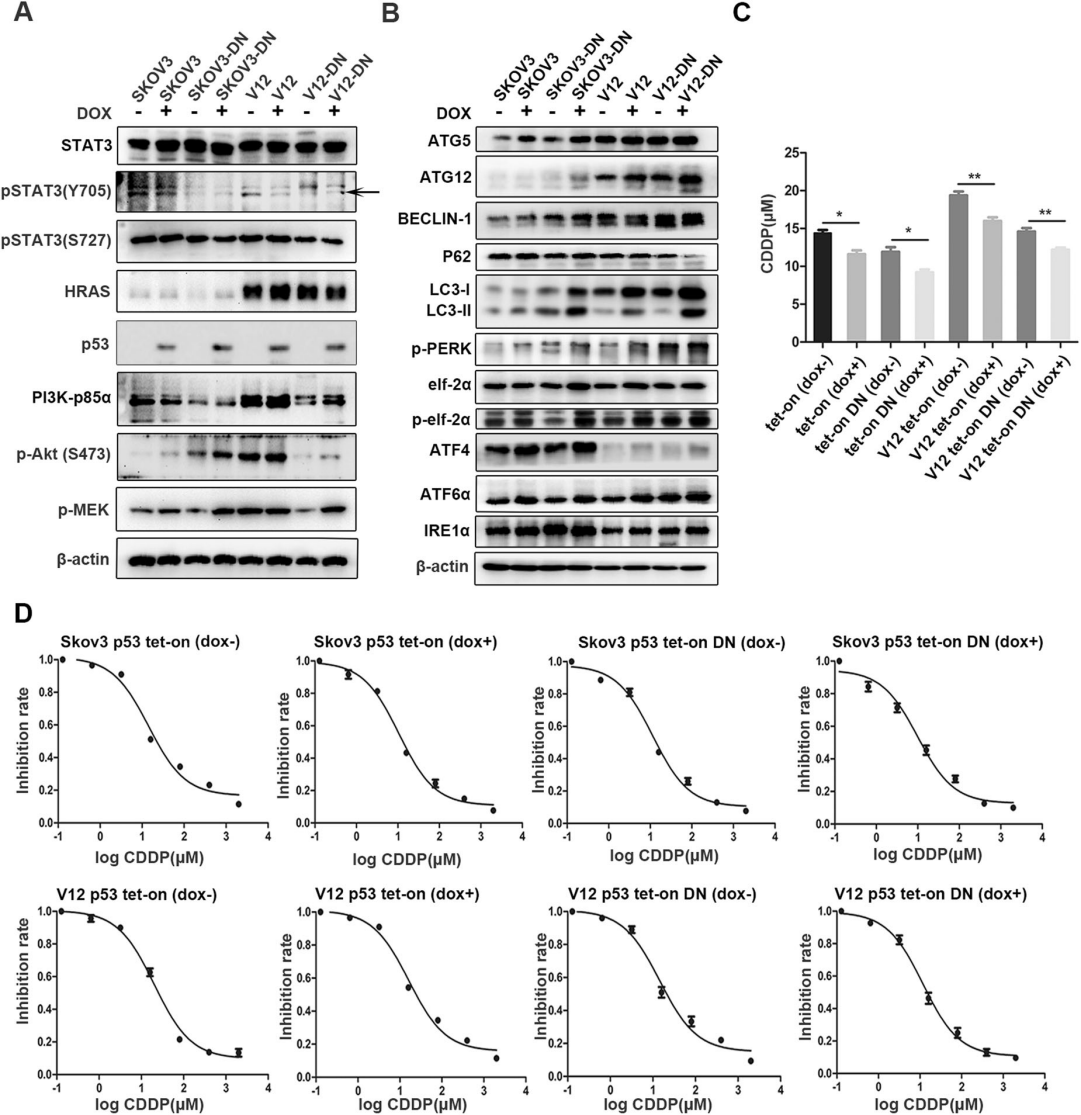
Examination of the crosstalk between STAT3 and p53/RAS signaling molecules and cisplatin resistance. **a** Analysis of pSTAT3 (S727), pSTAT3 (Y705), HRAS, p53, p-MEK, PI3K-p85α, and pAKT(S473) by western blot. **b** Analysis of autophagy-associated molecules and ER stress-associated molecules by western blot. **c**, **d** Sensitivity to cisplatin tested by MTT showing the IC50 values (**c**) and inhibition rates (**d**). *p < 0.05, **p < 0.01. Error bars = 95% CIs

The above results suggested that STAT3 might promote cisplatin resistance through inhibition of ERS-induced autophagy. Because p53 is commonly mutated and PI3K/AKT and MAPK-associated molecuels belong to RAS signaling^20,21^, we wondered if p53 and RAS are invovled in the STAT3-mediated autophagy, tumor growth, and cisplatin resistance. To investigate the effects of RAS and p53 on STAT3 function, we first introduced a dox-induction system to induce p53 expression in SKOV3 cells deficient with wild type p53, and then added a RAS mutant V12 active with both MAPK and PI3K/AKT into these cells afterwards^15^. The cDNA of STAT3-DN was also introduced into above cell lines (Fig. 6a). We found that pSTAT3 (Y705) but not pSTAT3 (S727) expression was inhibited by introduction of p53 only in RAS^V12^-transfected cells. RAS^V12^ increased PI3K-p85a/pAkt and pMEK, but did not increase pSTAT3, indicating that RAS and STAT3 are two independent molecules at the upstream of intracellular signaling. However, STAT3 inactivition highly suppressed the activation of the RAS downtream signaling molecules including PI3K/AKT and MEK. Surprisingly, wild-type p53 induction activated pAKT and pMEK mainly in STAT3-inactive cells (Fig. 6a). These results suggested that RAS and p53 might participate in the STAT3-induced autophagy through indirect regulation of the PI3K/AKT and MAPK signal pathways.

**Fig. 7:**
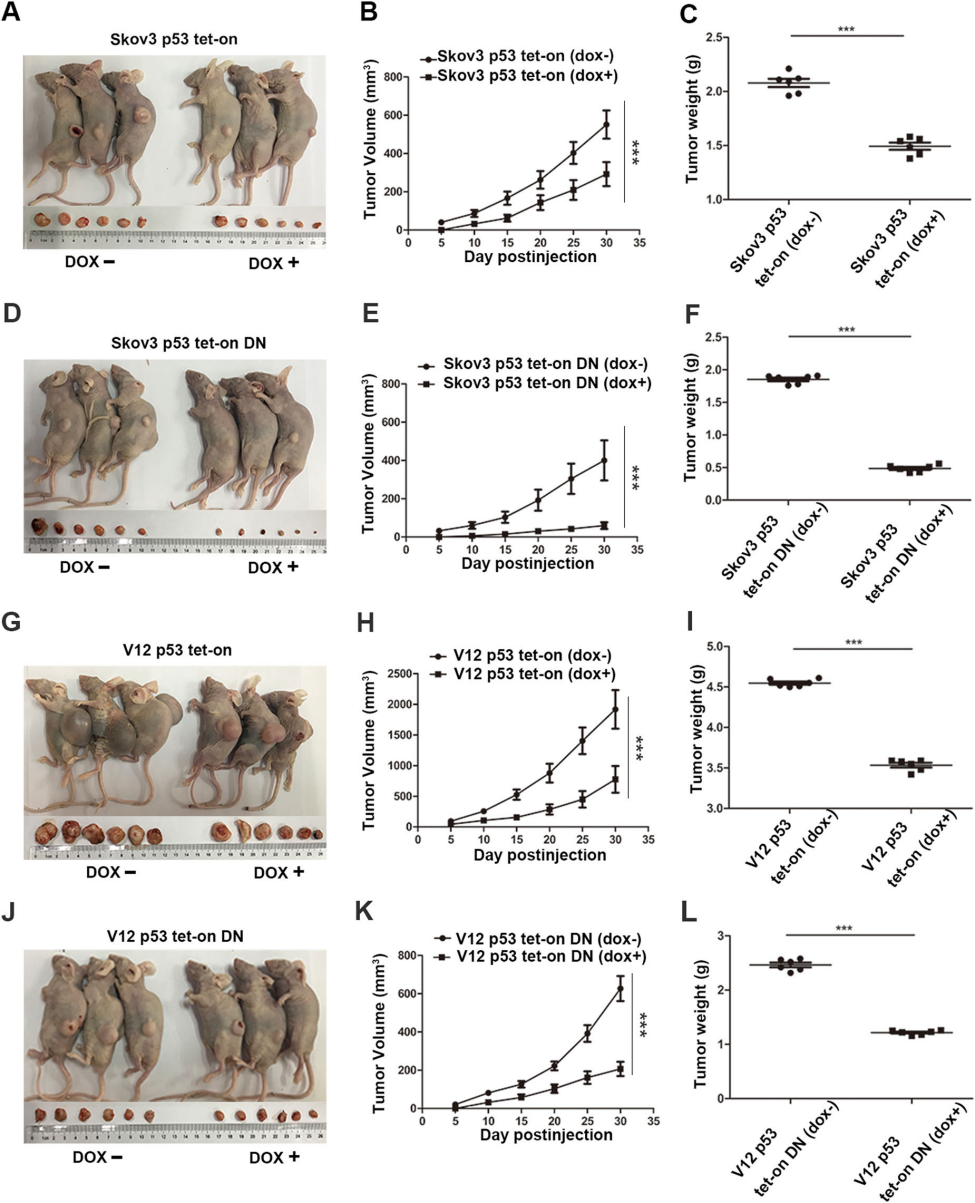
Xenograft tumor formation induced by cells with different status of STAT3, RAS and p53. **a**–**c** Induction of p53 in SKOV3 cells reduced animal tumor burden, tumor growth, and weights. **d**–**f** Introduction of STAT3-DN suppressed animal tumor burden, tumor growth, and weights, whereas induction of p53 in STAT3-inhibited cells further reduced the animal tumor burden, tumor growth, and weight. **g**–**i** Introduction of RAS^V12^ highly promoted the tumor growth and tumor weights, but induction of p53 in RAS^V12^ inhibited the tumor growth and weight. **j**–**l** Inhibition of STAT3 activity by transfection of STAT3-DN into RAS^V12^expressing cells remarkably blocked tumor growth and weight, whereas induction of p53 in these cells further decreased the tumor growth and weight. Error bars = 95% CIs. *n* = 6 mice/group

Further tests of molecules associated with ERS and autophagy showed that induction of p53 promoted the expression of ATG5, ATG12, BECLIN1, and LC3B, regardless of the presence or absence of RAS and STAT3-DN, although introduction of RAS^V12^strongly induced their expression (Fig. 6b). The ERS-related indicators including pPERK, pelf2α, ATF4, ATF6α were also increased by p53 introduction. IRE1α and ATF4 were highly inhibited by RAS^V12^, but did not interrupt the cellular autophagy induced by p53. These data suggest that both p53 and RAS strengthen the STAT3-DN-induced ERS and autophagy by activating the AKT/mTOR and MEK signaling. However, the cisplatin resistance of cells associated with p53 and RAS status was quite different because the inhibition of STAT3 without regard of RAS^V12^ reduced the IC50 of cells to cispaltin treatment and the induction of p53 with or without activation of RAS and STAT3 increased the sensitivity of the cells to cisplatin (Fig.6c, d). These data demonstrate that oncoproteins including RAS and STAT3 may confer chemoresistance through either induction or inhibition of autophagy, whereas tumor suppressors such as p53 may increase chemosensitivity through induction of autophagy.Animal experiments showed that the tumor growth rate was highly induced by RAS^V12^, but could be signlificantly inhibited by introduction of p53 and STAT3-DN (Fig. 7).

Based on above results, our study suggests that STAT3 controls ovarian cancer cisplatin resistance through the ERS-mediated autophagy and a crosstalk with p53 and RAS signaling molecules including MAPK and PI3K/AKT, which may be targeted along with cisplatin treatment for ovarian cancer.

## Discussion

According to literatures, the presence of constitutively activated STAT3 was found in ovarian cancer tissues and was reported to participate in a variety of cellular processes in ovarian cancer cells including proliferation, invasion, and metastasis^22,23^. Consistent with the current studies, our findings revealed that constitutively activated STAT3 promoted ovarian cancer cell proliferation, cell invasion, and metastasis, while the inhibition of STAT3 suppressed cell proliferation, cell invasion, and metastasis, most likely through the Slug/Snail-mediated regulation of the EMT markers such as E-cadherin and Vimentin.

Chemoresistance is an intricate problem in the treatment of ovarian cancer, which may also contribute to worse prognosis^24^. Recently, studies have found that autophagy could contribute to chemosensitivity through stimulated apoptosis pathway. In the current study, we revealed that overexpression of STAT3-C and STAT3 WT in OVCA429 cells activated the MEK/ERK and the PI3K/AKT/mTOR signaling pathways, which inhibited the ERS-associated molecules and blocked cellular autophagy, resulting in cisplatin resistance. In contrast, transfection of STAT-DN in SKVO3 cells repressed the MAPK and PI3K/AKT/mTOR signaling pathways, which activated the expression of ERS- and autophagy-associated molecules, leading to the reduced resistance of cells to cisplatin treatment. Moreover, cells with the different status of STAT3 also displayed the expression differences of the ERS- and autophagy-associated proteins upon cisplatin treatment, indicating that cancer cells may control their fate through ERS and autophagy in response to cisplatin treatment. Thus, targeting the associated molecules may provide a novel hint to improve cancer treatment efficacy.

The tumor suppressor p53 plays a major role in cell apoptosis, cell cycle arrest, DNA repair, and senescence. p53 is typically mutated in serous ovarian cancer and also associated with ovarian cancer chemoresistance^25–27^. Studies have shown that STAT3 and p53 regulate each other’s activities^28–30^. A recent study reported that a gain of function mutation in p53 inhibits STAT3-mediated tumor growth in colorectal cancer cells^31^. We identified that inhibition of STAT3 activated MAPK and PI3K/AKT/mTOR to stimulate autophagy, leading to reduced cisplatin resistance and tumor growth/metastasis, whereas induction of p53 decreased the expression level of STAT3 (Y705) and abolished the inhibition of pSTAT3 Y705 on the endoplasmic reticulum stress signaling pathway and autophagy signaling pathway, leading to upregulation of the ERS- and autophagy-associated indicators and the chemosensitivity of cells to cisplatin treatment. Moreover, induction of p53 in STAT3-inhibited cells with STAT3-DN transfection further promoted autophagy, which resulted in additional reduction of the cisplatin resistance of cancer cells and the tumor growth. Thus, these data suggest that an inverse regulation of autophagy between p53 and STAT3 carries out opposite effects on ovarian cancer metastasis and cisplatin resistance, and that p53 and STAT3 may have a crosstalk through the collaborative regulation of MAPK and PI3K/ATK signaling.

RAS could serve as the upstream of the PI3K/AKT and MEK/ERK signaling pathways to regulate tumor development^32^. Studies have shown that, at the upstream of the PI3K/AKT signaling pathway, RAS may downregulate BECLIN1 by activating PI3K/AKT to inhibit autophagy^33,34^; however, RAS can also induce autophagy through the MEK/ERK signaling pathway^35^, which have been confirmed by a recent study from our research group^15^. Because RAS^V12^ is supposed to be active in both PI3K/AKT and MAPK signaling, we wondered whether the oncogenic RAS also participates in STAT3-induced autophagy, chemoresistance, and tumor growth. In vitro experiments showed that introduction of RAS^V12^ activated both MEK and PI3K/AKT pathways to lift ERS- and autophagy-associated molecules, granting cancer cell resistance to cisplatin treatment. However, introduction of a STAT3 dominant-negative mutant (STAT3-DN) in RAS^V12^ further promoted the expression of the ERS and autophagy markers, but the MAPK and PI3K/AKT signaling molecules were highly suppressed. Thus, RAS and STAT3 consistently contribute to ovarian tumor growth, metastasis, and cisplatin resistance, but inversely regulate the MAPK- and PI3K/AKT-mediated ERS and autophagy. Therefore, p53, STAT3, and RAS may inhibit or promote ovarian cancer cell tumor growth, metastasis, and chemoresistance through the differential regulation of the MAPK- and PI3K/AKT-mediated ERS and autophagy. To improve the chemotherapeutic efficacy of ovarian cancer, p53, STAT3, RAS, and the ERS/autophagy-associated molecules should all be considered and targeted accordingly.

## Conclusions

Our data suggest that ovarian cancer metastasis and cisplatin resistance is associated with the differential regulation of the MAPK/PI3K/AKT-mediated ERS and autophagy through the crosstalk of STAT3 and p53/RAS signaling.
